# Support From the Top: Diverse Leadership Partners for Sustained STAR-VA Implementation

**DOI:** 10.1093/geroni/igab046.2065

**Published:** 2021-12-17

**Authors:** Michele Karel, A Lynn Snow, Christine Hartmann, Jenefer Jedele, Lisa Minor

**Affiliations:** 1 Veterans Health Administration, Washington, District of Columbia, United States; 2 Tuscaloosa VA Medical Center, Tuscaloosa, Alabama, United States; 3 VA Bedford Healthcare System, VA Bedford Healthcare System, Massachusetts, United States; 4 Veterans Health Administration, Ann Arbor, Michigan, United States; 5 Geriatrics and Extended Care, Washington, District of Columbia, United States

## Abstract

The STAR-VA program was an initiative out of what is now called the VA Office of Mental Health and Suicide Prevention, partnering with the national Offices of Geriatrics and Extended Care and Nursing Services. Ongoing collaboration with these national, as well as regional and medical-center-level leaders, has been critical for informing program implementation and dissemination strategies. We will discuss several key partnered strategies, including (1) linking STAR-VA to national CLC systematic quality improvement efforts; (2) engaging national inter-office program leaders in decisions about outreach to and inclusion of facilities in STAR-VA training and implementation; (3) training local STAR-VA champions on strategies for engaging local leadership support; (4) briefing leaders across the system with program updates; and (5) using national VA data to inform STAR-VA sustained implementation. Discussion will address challenges and opportunities for engaging leadership stakeholders in facilitating sustained implementation of evidence-based programs.

